# Drugs in the Treatment of Disease

**Published:** 1863-11

**Authors:** 


### EDITORIAL DEPARTMENT.

### DRUGS IN THE TREATMENT OF DISEASE.

The element of physic in medical practice is certainly becoming more simple; our drugs are fewer and much less complicated. The progress of true medical science has greatly qualified our estimate of the value of mere medicine in the treatment of disease. While physicians regard quinine as cure for ague—nobody knows how; and many believe that mercury has some strange power over venereal poison; yet even quinine often fails, and the uses of mercury are becoming circumscribed rather than extending. Every day is showing the value of measures founded upon a rational study of the body and its diseases; at the same time, no sound man is very sanguine in his expectations of discovering specifics for the cure of the various diseases common among mankind.

The sore that used to be treated with an unguent composed of twenty ingredients, heals under moist lint, when placed in proper position, or supported by the stimulus of gentle pressure. The pneumonia that used to be attacked with heroic remedies—bleeding, antimony and calomel—now gets well with horizontal position and small doses of Dover's powder. Inflammation even of the serous membranes, which formerly received most active medication is now observed to terminate favorably, if pain is abated and sleep obtained by a full anodyne. The more painless or even pleasant a physician can make his treatment, the more he can divest it of irritating and disturbing characters, the better is it, and the greater and more acceptable is he. The chief characteristic of advancing therapeutics, is to respectfully watch the natural course of disease, to regard pathological processes only as modifications of physiological ones, with natural tendency to terminate in harmonious and healthy action when the obstructions are overcome which these pathological processes themselves were put in action to remove. We often see in the worst forms of disease "an effort of nature to throw off the morbific matter, and thus cure the patient." All this is done without any detraction from the dignity and importance of the physician; he is indeed much more worthy public admiration and confidence than he who would attain no more than the same result by the most active medical warfare.

Physicians never talked so modestly about "curing" disease as now, and those who excel in this modesty do most towards the furtherance of this object.

Pseudo-medicine talks loudly of specifics, and makes no account of the provisions of nature for the cure of disease. Medicine is everything, nature perverse and destructive without its regenerating power, "every disease and symptom of disease has its corresponding remedy," we may say remedies," for it is astonishing how multiplied and unending are its resources. One medicine is never sufficient to cure disease, it always requires two or more, alternated with exactness, taken in equal quantities and with equal frequency. Upon this plan the poor victim of the "infinitesimal" it is said often becomes the receptacle of a "most unprincipled amount of physic."

Perhaps it may be well to suggest, that medicine should be so studied and taught as to make more apparent the "great gulf" which is fixed between regular scientific practice and the various quackeries of the day. There is no field of operation where the distinction is more real between intelligent, honest and progressive medicine, and the various forms of empiricism and quackery, than this open ground of constant unlimited and unending medication. Medicine should be so used that the most unobserving patient will perceive, that it is only one of many means to an end, assisting in the performance of the great functions of nature, and acting not mysteriously, but naturally and rationally. Above all he should understand that the duty of the physician is not mainly to advise the use of drugs, that he pays not for physic, but for the advice, attention, skill and enlightened judgment of his medical attendant.

Medicine-monger is a term not yet applicable only to those who practice the various forms of quackery, but in too many instances it is descriptive of physicians who from habit and extensive practice, have not stopped to sufficiently consider the value of other and more rational means of cure; and still continue in the administration of medicine which does no good, and often protracts disease, and produces much harm. In extenuation of this practice it is often said, that community is not yet sufficiently educated to accept the advice of a physician without medicine; this is in some degree true, but sensible patients will soon learn to prize and properly appreciate the advice of an honest physician, who fearlessly and unconditionally tells him, you have a disease which medicine has no power to control; its use will make you no better, and is liable to do you great harm. We suppose that if physicians should confine themselves to prescribing medicine only where it is plainly indicated, and should advise food, clothing, exercise and other hygienic conditions when these would answer the indications equally well, or even better, that medicine proper would meet with a vastly diminished sale, and that the glory and immortal honor of the profession would be fully established. The habit of giving some medicine, in almost all cases, and of giving two or three different kinds, in a great many, has really become established with the majority of practitioners. While we do not propose to intimate that the compounds given are usually very injurious, or in any way capable of doing great harm, we yet claim that they are unnecessary, sometimes injurious, and always objectionable, unless plainly indicated. It requires much less time and thought to write a prescription, than it does to explain the reasons why no prescription is necessary. Many patients might at first be as well satisfied, but in the end the true greatness of the physician who does his duty, will appear.

Treating disease with only the medicine which is useful, is certainly no easy task, or one which the younger members of the profession will find possible for them to inaugurate. The older and fully established can give opinions which are authority, while the young physician must be not only a hero, but also a martyr if he use fewer weapons.

Disease is almost everywhere over-treated, and nothing can be more plain or more easily demonstrated than this proposition. If we did not know it was true we should be glad to speak otherwise. It did appear at one time that the vagaries of Hahnneman were to be adopted in degree to prevent somewhat the injurious abuse of medicine, but even the belief that Homœopathic remedies would at least do no harm, has long since been dissipated with the knowledge that even the disciples of this monstrous delusion are drugged more extensively and more dangerously than any other; they are literally "fed on drugs," in doses which would do honor to the chivalrous days of "heroic practice."*

It is not that we would lessen faith in the value of judicious medication, or detract in the least from the confidence which the intelligent physician reposes in his therapeutics; but we would rather increase his expectation of usefulness by excluding from his armament the useless weapons, and suggesting the employment of the remaining ones only when necessity demands. That there is, however, too much confidence in the influence of medicine, and too great readiness to attribute results to its effects, there can be no doubt; this holds good both with physicians and patients, still the responsibility rests mostly with the profession. If those who minister at the altar of medical practice, hold to, and often express erroneous views, the disciples who attend its ministrations will imbibe its error, and transmit it, greatly augmented, to brother followers. We sometimes wonder at the ignorance of communities and individuals in matters pertaining to our profession; we can only say, "like priests, like people."

---

* A recent visit to a chlid that had been treated nine days for "cerebral congestion," and given up to die by Homœopathic physicians, terminated in an expression of opinion that the child had no disease whatever, except the sugar granules. Parents incredulous, but consented to suspension of all medicine for twenty-four hours, at termination of which period, child was playful and well as ever. Comment unnecessary,

When a few years since the people first thirsted for some medical knowledge and discovered that the physician was not the medium of strange spiritual or superhuman influence, but worked by tangible and natural agencies, the dignity of physicians was so august, that to tell patients the name of the medicine they were to use, was regarded an unbecoming condescention; while inquiry upon any such point was often met by indignant intimations of very improper interference. This haughty and silent reserve could be preserved only for a short period, and was finally construed to prove that the medicine was always "calomel and jalap," and that the doctor was determined by every means possible to force its acceptance. Now came the revolt, and to meet the necessities of the case, vegetable doctors and rapidly succeeding ics and isms, until the world is full of imposture in medicine. For this we are sorry, but it is not so much our concern that the masses of mankind are ignorant and stupid in these matters, as that the members of the profession should in any way contribute to the result.

To reform and educate the masses, to a just appreciation of the value of medicine as such, or to the importance of observing the plainest and most common hygienic rules and conditions may well be looked upon as a hopeless task. Intelligent men in other matters will astonish us with their ignorance and stupidity in matters of medicine, and when the various forms of absurdity which now receive credence shall have been exploded and passed away, others will not be wanting to take their places equally absurd and preposterous. About one-quarter of the people follow after and take naturally to the systems of empiricism as they severally make their appearance. Thomsonian, Eclectic, Galvanic, Homœopathic, Magnetic, Spirltualistic, Dermotic, Aquatic, etc., each in turn receives their support, and is the system they advocate. They are worthy people; in most respects they have not less common sense and observation than others, but are monomaniac in matters pertaining to their health. That this is the true condition of things is no disadvantage to the capable physician; on the other hand we regard it as a blessing, since truthful and honest advice, which had a show of consistency and probability about it, would be repudiated by them as too dull and old fashioned for their faith, having nothing wonderful, mysterious or unaccountable about it, nothing to attract or confound. Physicians do not want such patients upon any terms of remuneration, and it cannot be regarded otherwise than a favor that their wants can be supplied from the ever open fountains of delusion.

A more careful study of the natural history of disease, and a more thorough acquaintance with the conditions of its progress and decline, will enable us to estimate the true value of our remedies, and prevent in a great degree the administration of medicines which can do nothing better than amuse the sick while nature restores the health. This, however, is not the true view to take of unnecessary medication. It is an unwarrantable deception and fraud; it lowers the true nobility of the profession, and gives it a near alliance to quackery.

